# HLA-DR Expression Level in CD8^+^ T Cells Correlates With the Severity of Children With Acute Infectious Mononucleosis

**DOI:** 10.3389/fimmu.2021.753290

**Published:** 2021-11-03

**Authors:** Yun Wang, Ying Luo, Guoxing Tang, Renren Ouyang, Minxia Zhang, Yuhuan Jiang, Ting Wang, Xiwen Zhang, Botao Yin, Jin Huang, Wei Wei, Min Huang, Feng Wang, Shiji Wu, Hongyan Hou

**Affiliations:** ^1^ Department of Laboratory Medicine, Tongji Hospital, Tongji Medical College, Huazhong University of Science and Technology, Wuhan, China; ^2^ Department of Clinical Laboratory, First Affiliated Hospital of Nanchang University, Nanchang, China

**Keywords:** EBV, infectious mononucleosis, CD8^+^ T cells, CD4^+^ T cells, HLA-DR^+^ CD8^+^ T cells

## Abstract

**Background:**

This study aimed to assess the host immune signatures associated with EBV infection and its clinical value in indicating the severity of children with acute infectious mononucleosis (IM).

**Methods:**

Twenty-eight pediatric patients with IM aged 3–8 years were enrolled. The immune phenotypes and cytokine secretion capability of T cells were detected.

**Results:**

The percentages and absolute numbers of CD3^+^ and CD8^+^ T cells were significantly increased in IM patients compared with HCs. The percentages of Naïve CD4^+^ and CD8^+^ T cells were decreased but with increased percentages of memory CD4^+^ and CD8^+^ T subsets. Our results showed the upregulation of active marker HLA-DR, TCR-αβ, and inhibitory receptors PD-1, TIGIT in CD8^+^ T cells from IM patients, which suggested that effective cytotoxic T cells were highly against EBV infection. However, EBV exposure impaired the cytokine (IFN-γ, IL-2, and TNF-α) secretion capability of CD4^+^ and CD8^+^ T cells after stimulation with PMA/ionomycin *in vitro*. Multivariate analysis revealed that the percentage of HLA-DR^+^ CD8^+^ T cells was an independent prognostic marker for IM. The percentage of HLA-DR^+^ CD8^+^ T cells was significantly correlated with high viral load and abnormal liver function results.

**Conclusion:**

Robust expansion and upregulation of HLA-DR in CD8^+^ T cells, accompanied with impaired cytokine secretion, were typical characteristics of children with acute IM. The percentage of HLA-DR^+^ CD8^+^ T cells might be used as a prominent marker not only for the early diagnosis but also for indicating the severity of IM.

## Introduction

Infectious mononucleosis (IM) is an acute infectious disease in children caused mainly by Epstein-Barr virus (EBV) infection (1). Epidemiological studies showed that the positive rate of EBV is estimated to be more than 90% worldwide (2). The typical clinical manifestations of children with IM include fever, sore throat, lymph node enlargement, hepatosplenomegaly, skin rash, eyelid edema (3, 4). However, the initial symptoms of IM are variable and lack specificity. Although IM presents as a self-revolving illness, some patients may develop serious complications, such as spleen rupture, malignancies, and EBV-related hemophagocytic syndrome (HPS) (5–7). Therefore, accurate early diagnosis, timely treatment, and effective monitoring are important for children with acute IM.

The host immune system exerts a key function in the recognition and elimination of EBV infection, and the dysregulation of immune response contributes to the development and progression of disease (8). EBV preferentially infects B lymphocytes and results in the establishment of lifelong latent infection of the host (9). It is generally believed that these cells are rigorously controlled *in vivo* by cytotoxic T cells (CTLs) (10). Inconsistent with this, a robust T cell response is specific for lytic and latent cycle viral antigens (11, 12). The intensive proliferation of CD8^+^ T with typical form plays a critical role in the clearance of EBV infection, which also explains the reduced ratio of CD4/CD8 lymphocytes (13). Moreover, the depletion of CD8^+^ T cells results in increased viral load and IM-like EBV infection in mice (14). Further studies demonstrated the importance of T cells and NK cells in controlling EBV infection in certain patients with immuno-deficiencies (15, 16). Therefore, a deep investigation of EBV-associated host immune response in children with acute IM is necessary for understanding the pathogenesis of EBV and EBV-related diseases.

In this study, we compared the differences of lymphocyte subsets between IM patients and healthy controls (HCs). The subpopulations of Naïve, memory/effector cells, the expression levels of active marker HLA-DR, co-stimulatory molecule CD28, T cell receptors (TCR-αβ, TCR-γδ), inhibitory receptors, and the cytokine secretion capabilities of CD4^+^ T and CD8^+^ T cells were evaluated in pediatric patients at the diagnosis of IM. Then CD8^+^ T cells were subdivided into CD8^high^ and CD8^dim^ T cell populations depending on the intensity of fluorescence, and the differences of phenotypes between the two cell populations were compared. In addition, the subtypes of B cells and monocytes were also detected. Our study provided a comprehensive evaluation of mononuclear cells in peripheral blood, which is critically important for further elucidating the pathogenesis mechanisms during EBV infection.

## Materials and Methods

### Patients

A total of 28 pediatric patients (19 males and 9 females) diagnosed with IM were included in this study from January to June 2021. None of these patients had previous evidence for an underlying immunodeficiency, and none of them received previous immunosuppressive drugs. In addition, 18 HCs (9 males and 9 females) were also recruited and determined by interview and physical examination. The exclusion criteria include HIV and HCV positive. This study was approved by the ethical committee of Tongji Hospital, Tongji Medical College, Huazhong University of Science and Technology. All of the subjects’ legal guardian/next of kin provided written informed consent for their participation in this study.

### TBNK Lymphocyte Counting

The percentages and absolute numbers of CD4^+^ T cells, CD8^+^ T cells, B cells, and NK cells were determined using TruCOUNT tubes and BD Multitest 6-color TBNK ReagentKit (BD Biosciences) according to the manufacturer’s instructions. In brief, 50 μl of whole blood was labeled with six-color TBNK Ab cocktail regent for 15 min at room temperature. After adding 450 μl of FACSlysing solution, samples were analyzed with FACSCanto flow cytometer using FACSCanto clinical software (BD Biosciences).

### Lymphocyte Phenotypes, B Cell and Monocyte Subsets Analysis

Heparinized venous blood was collected from IM patients and HCs. The following monoclonal antibodies and reagents were added to 100 µl whole blood for the analysis of lymphocyte phenotypes. The antibodies in tube 1 were anti-CD45-PerCP (BD phamingin, 2D1), anti-CD3-APC-H7 (BD phamingin, SK7), anti-CD4-V450 (BD phamingin, RPA-T4), anti-CD8-PE/Cy7 (BD phamingin, SK1), anti-CD28-PE (BD phamingin, L293), and anti-HLA-DR-APC (BD phamingin, L243). The antibodies in tube 2 were anti-CD45-PerCP (BD phamingin, 2D1), anti-CD3-APC-H7 (BD phamingin, SK7), anti-CD4-BV510 (BD phamingin, SK3), anti-CD45RA-FITC (BD phamingin, L48), anti-CD8-PE/Cy7 (BD phamingin, SK1), anti-CCR7-PE (BD phamingin, 3D12), anti-CD25-APC (BD phamingin, 2A3), and anti-CD127-BV421 (BD phamingin, HIL-7R-M21). The expression of TCR on T cells was detected using anti-CD45-PerCP (BD phamingin, 2D1), anti-TCR-αβ-FITC (BD phamingin, WT31), anti-TCR-γδ-PE (BD phamingin, 11F2), anti-CD3-APC/H7 (BD phamingin, SK7), anti-CD4-V450 (BD phamingin, RPA-T4), and anti-CD8-APC (BD phamingin, SK1). The subsets of B cells were detected using anti-CD38-FITC (BD phamingin, HB7), anti-CD19-PE/Cy7 (BD phamingin, SJ25C1), anti-CD27-PerCP (BD phamingin, 2D1) and CD45-V500C (BD phamingin, 2D1), and anti-IgD-APC (BD phamingin, IA6-2). The subsets of monocytes were detected using anti-HLA-DR-FITC (BD phamingin, L243), anti-CD16-PE (BD phamingin, B73.1), anti-CD14-APC (BD phamingin, MFP9), and anti-CD45-V500C (BD phamingin, 2D1). The expressions of inhibitory receptors on T cells were assessed using CD45-V500C (BD phamingin, 2D1), anti-CD4-APC-H7 (BD phamingin, RPA-T4), anti-CD8-PE/Cy7 (BD phamingin, SK1), anti-PD-1-V450 (Biolegend, EH12.2H7), and anti-TIGIT-PE (Biolegend, 15153G). Isotype controls with irrelevant specificities were included as negative controls. All of these cell suspensions were incubated for 20 min at room temperature. After lysing red blood cells with lysing solution, the cells were washed and re-suspended in 300 μl PBS. The cells were then analyzed with FACSCanto flow cytometer.

### The Capability of Cytokine Secretion Analysis

Peripheral blood mononuclear cells (PBMCs) were isolated from heparinized venous whole blood by Ficoll-Hypaque density gradient centrifugation. In order to measure intracellular cytokines, PBMCs (2.5 × 10^5^) were seeded in 96-well plates in 100 µl of IMDM medium and stimulated with Leukocyte ActivationCocktail (Becton Dickinson GolgiPlug) for 4 h. After stimulation, the cells were collected and stained with the following monoclonal antibodies: anti-CD45-PerCP (BD phamingin, 2D1), anti-CD3-APC/H7 (BD phamingin, SK7), anti-CD4-V450 (BD phamingin, RPA-T4), and anti-CD8-PE/Cy7 (BD phamingin, SK1), followed by fixation and permeabilization, and staining with intracellular anti-IFN-γ-APC (BD phamingin, B27) antibody, anti-TNF-α-FITC (Biolegend, MAb11), and anti-IL-2-PE (Biolegend, MQ1-17H12). The cells were then analyzed with FACSCanto flow cytometer.

### Statistical Analysis

The results are presented as mean ± standard deviation (SD), or as median with interquartile range (IQR) when appropriate. Continuous variables were compared with Mann-Whitney U-test. Spearman’s rank correlation test for non-parametric data was employed to analyze the relationship between two factors. Heatmaps were generated using R3.2.3 (R Foundation for Statistical Computing) with the pheatmap package 48. All variables with statistical significance were taken as candidates for multivariable logistic regression analyses. Receiver operating characteristic (ROC) curve analysis was performed to ascertain the optimal cutoff value of parameters associated with maximum sensitivity and specificity. Statistical analyses were performed using GraphPad Prism version 8 (San Diego, CA, USA) and SPSS version 22.0 SPSS, Chicago, IL, USA). Statistical significance was determined as *p* < 0.05.

## Results

### Participant Characteristics

The present study recruited 28 children diagnosed with IM, including 19 (67.9%) males and 9 (32.1%) females, median age of 6.0 (25^th^–75^th^ quartiles, range 3.9–7.75 years). The most common clinical manifestations of the IM patients were enlarged tonsils (67.9%), pharyngeal hyperemia (64.3%), fever (60.7%), cervical lymphadenopathy (53.6%), and eyelid edema (46.4%). The clinical characteristics and laboratory test results of the included subjects are presented in **Table 1**.

**Table 1:** Basic characteristics of pediatric patients with infectious mononucleosis.

	IM (n=28)	HC (n=18)	*p* value
**Age (years)**	6.0 (2.9-7.75)	5.58 (3.75-7.25)	0.942
**Gender**			
Male	19 (67.9%)	9 (50%)	
Female	9 (32.1%)	9 (50%)	0.354
**Clinic mainfestations**			
Enlarged tonsills	19 (67.9%)		
Pharyngeal hyperemia	18 (64.3%)		
Fever	17 (60.7%)		
Cervical lymphadenopathy	15 (53.6%)		
Eyelid edema	13 (46.4%)		
Sleep snoring	11 (39.3%)		
Nasal obstruction	8 (28.6%)		
Pharyngalgia	5 (17.9%)		
Sniffles	5 (17.9%)		
Cough	2 (7.1%)		
**Laboratory test**			
Atypical lymphocytes (>10%)	6 (21.4%)		
Plasma EBV-DNA (copies/mL)	10 (35.7%)		
EA IgG (U/mL)	16 (57.1%)		
VCA IgG (U/mL)	21 (75.0%)		
EBV IgM (U/mL)	26 (92.9%)		
EBNA IgG (U/mL)	9 (32.1%)		
WBC (×10^9^/mL)	14.06±5.29	7.10±0.69	<0.001
Neutrophils (×10^9^/mL)	3.14±0.37	3.32±0.43	0.508
Lymphocytes (×10^9^/mL)	9.44±0.64	3.36±0.35	<0.001
Platelets (×10^9^/mL)	228.0±15.11	315.9±16.1	0.001
Hemoglobin (g/L)	121.8±1.48	128.1±1.50	0.006
ALT (U/mL)	104.5±22.67	14.35±1.36	<0.001
AST (U/mL)	95.29±17.93	23.71±1.31	<0.001
LDH (U/mL)	495.5±156.7	214.1±32.81	<0.001
**Average days from symptom onset to sample collection**	6.0 (4.0-8.0)		

Data are presented as number of patients (%) or median (25th-75th).

IM: EBV: Epstein-Barr Virus, VCA: viral capsid antigen, EA: early antigen, EBNA: EBV nuclear antigen. ALT: alanine aminotransferase, AST: aspartate aminotransferase, LDH: lactate dehydrogenase.

### Characterization of Lymphocyte Subsets and Differential Phenotypes of Memory T Cells in Peripheral Blood

**Figure 1:**
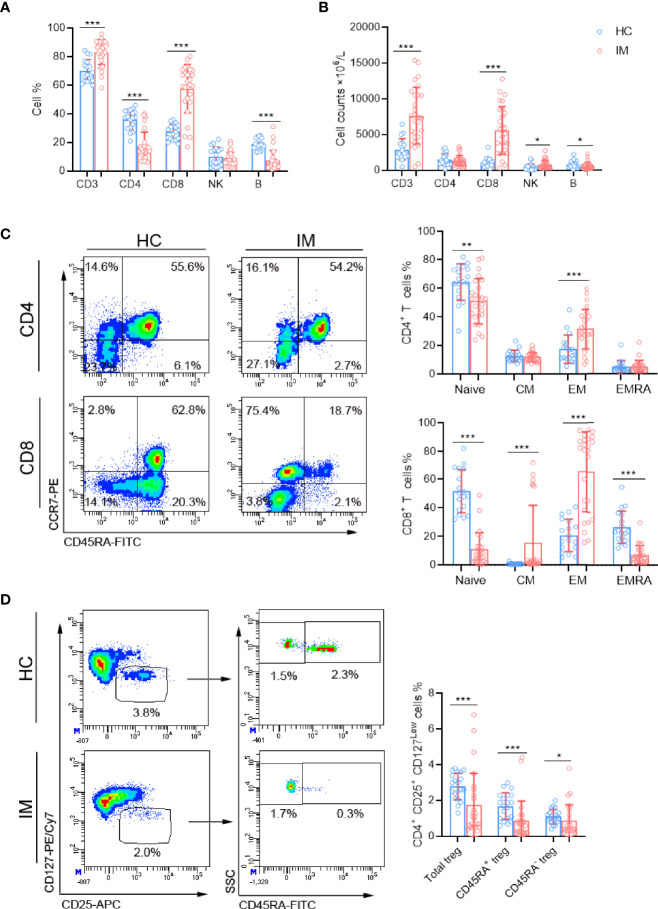
The lymphocyte subsets and immunophenotype characteristics. Circulating lymphocytes in pediatric patients newly diagnosed with infectious mononucleosis (IM) and healthy controls (HCs) were analyzed using flow cytometer. **(A, B)** The percentages and absolute numbers of T cells, B cells, and NK cells in different groups were expressed as mean with standard deviation (SD). **(C)** A typical example of the gating strategy is shown. CD4^+^ and CD8^+^ Naïve (T_Naïve_, CCR7^+^ CD45RA^+^), central memory (T_CM_, CCR7^+^ CD45RA^−^), effector memory (T_CM_ CCR7^−^ CD45RA^−^), and EMRA (T_EMRA_, CCR7^−^ CD45RA^+^) T cell subsets were shown. The percentages of CD4^+^ T and CD8^+^ T cell subtypes in different groups were expressed as mean with SD. **(D)** Representative flow dot plots showing the gating of Tregs (CD4^+^ CD25^+^ CD127^low^), CD45RA^+^ Tregs and CD45RA^−^ Tregs. The percentages of Tregs, CD45RA^+^ Tregs, and CD45RA^−^ Tregs in lymphocytes were expressed as mean with SD. Blue circle points represent HCs, and red circle points represent IM patients. *p < 0.05, **p < 0.01, ***p < 0.001.

The lymphocyte subsets in peripheral blood were analyzed and revealed that the percentages and absolute numbers of CD3^+^ T and CD8^+^ T cells were significantly increased in IM patients compared with that of HCs. The percentages of CD4^+^ T cells were relatively decreased but with no significant difference in absolute cell counts. Meanwhile, elevated numbers of NK cells were observed in IM patients but with decreased percentages and numbers of B cells (**Figures 1A, B**). The expression of CCR7 and CD45RA were used to distinguish the memory phenotypes of T cells (**Figure 1C**) (17). The results showed the percentages of CD4^+^ and CD8^+^ T Naïve subtype (T_Naïve_, CCR7^+^ CD45RA^+^) in the IM group were decreased, and concomitantly with accumulated percentages of CD4^+^ and CD8^+^ T effector memory subtype (T_EM_, CCR7^−^ CD45RA^−^) compared with that of HCs. The percentage of CD8^+^ T central memory subtype (T_CM_, CCR7^+^ CD45RA^−^) was increased, but the percentage of terminally differentiated effector CD8^+^ T subtype (T_EMRA_, CCR7^−^ CD45RA^+^) was decreased (**Figure 1C**). Furthermore, the proportions of regulatory T cells (Tregs, CD4^+^/CD25^+^/CD127^low^), including CD45RA^+^ Naïve Treg and CD45RA^−^-induced Treg, were all significantly decreased in patients with IM than that of HCs (**Figure 1D**).

### CD4^+^ and CD8^+^ T Cells Exhibited Highly Activated Phenotypes in IM Patients

**Figure 2:**
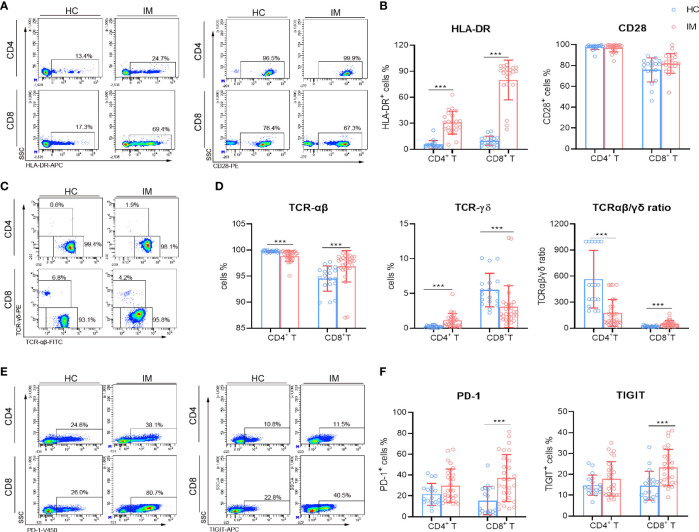
Immunophenotype characteristics of T cells in IM patients. **(A, B)** Representative flow cytometry gating strategy for the expression of HLA-DR and CD28 in T cells. The percentages of HLA-DR and CD28 positive cells in CD4^+^ and CD8^+^ T cells from IM patients and HCs were expressed as mean with SD. **(C)** Representative dot plots showing the expression of TCR in CD4^+^ and CD8^+^ T cells. **(D)** The percentages of TCR-αβ and TCR-γδ positive cells and the ratio of TCR-αβ and TCR-γδ in CD4^+^ and CD8^+^ T cells were expressed as mean with SD. **(E)** Representative dot plots showing the expression of PD-1 and TIGIT in CD4^+^ and CD8^+^ T cells. **(F)** The percentages of PD-1 and TIGIT positive cells were expressed as mean with SD. Blue circle points represent HCs, and red circle points represent IM patients. ***p < 0.001.

The immune-phenotypes of CD4^+^ and CD8^+^ T cells were determined through detecting the expression of activation, co-stimulatory, TCR, and inhibitory molecules. IM patients showed obviously high levels of HLA-DR expression in CD4^+^ and CD8^+^ T cells, but the expression of CD28 showed no significant difference (**Figures 2A, B**). Decreased expression of TCR-αβ and increased expression of TCR-γδ were observed in CD4^+^ T cells, whereas converse results were observed in CD8^+^ T cells. The ratio of TCR-αβ and TCR-γδ in CD4^+^ T cells was decreased but increased in CD8^+^ T cells (**Figures 2C, D**). CD8^+^ T cells in IM patients also displayed significantly high levels of PD-1 and TIGIT, but not for CD4^+^ T cells (**Figures 2E, F**). Furthermore, IM patients were stratified into two groups according to age (age 0–5 years and 6–13 years), and the lymphocyte numbers and phenotypes were analyzed. Our results showed that a higher percentage of CD3^+^ T cells but lower counts of CD4^+^ T cells and B cells were observed in older children than that of younger children (**Supplementary Figures 1A, B**). More percentages of naive CD8^+^ T cells were differentiated into EM subsets in older children (**Supplementary Figures 1C, D**). However, the expression of CD28 and HLA-DR showed no significant difference between the two age groups (**Supplementary Figures 1E, F**). Notably, an increased proportion of CD8^dim^ T cells along with robust CD8^high^ and CD8^dim^ T cell expansion were found in IM patients, and comparisons of active phenotypes between CD8^high^ and CD8^dim^ T cells were performed (**Supplementary Figures 2A, B**). Increased levels of HLA-DR and TCR in CD8^high^ and CD8^dim^ T cells and a high level of CD28 in CD8^high^ T cells were observed in IM patients than HCs, but no significant differences of HLA-DR and CD28 were found between the two subpopulations (**Supplementary Figures 1C, D**). These data confirmed the robust expansion and highly active status of CD8^+^ T cells in response to EBV infection.

### CD8^+^ T Cells From IM Patients Showed Impaired Function of Cytokine Secretion

**Figure 3:**
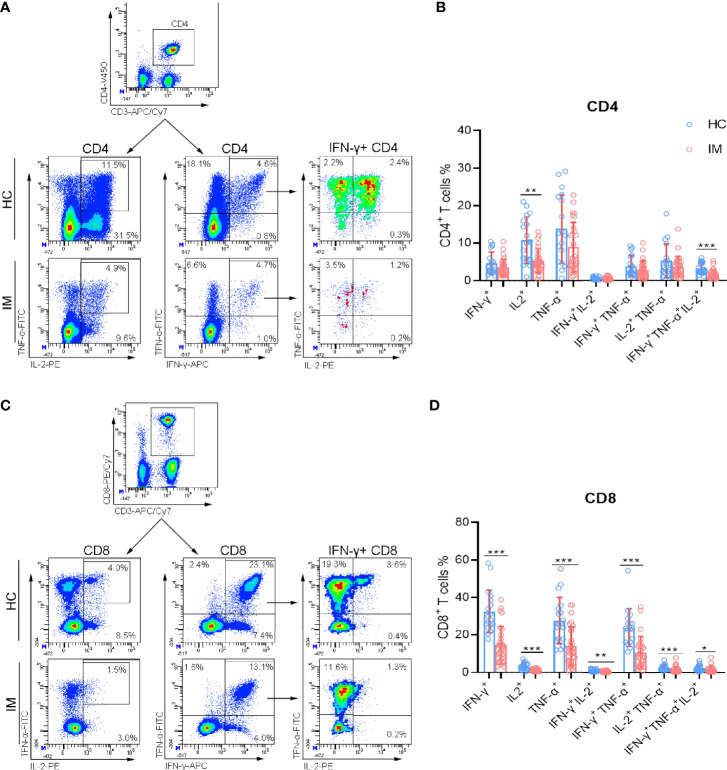
The cytokine secretion capability of T cells. PBMCs isolated from IM patients, and HCs were stimulated with PMA/ionomycin. After culture, the cells were collected and the production of intracellular IFN-γ, TNF-α, and IL-2 in CD4^+^ and CD8^+^ T cells was analyzed by flow cytometry. **(A)** Representative dot plots showing the gating strategies for intracellular cytokines in CD4^+^ T cells. CD4^+^ T cells were shown, and the percentages of IL-2, IFN-γ, and TNF-α single-positive, IL-2^+^ TNF-α^+^ and IFN-γ^+^ TNF-α^+^ double-positive cells were gated. Then IFN-γ single-positive cells were shown, and IFN-γ^+^ IL-2^+^, IFN-γ^+^ TNF-α^+^ IL-2^+^ cell populations were gated. **(B)** The percentages of IFN-γ, TNF-α, and IL-2 single-, double-, and triple-positive cells within CD4^+^ T cells from IM patients and HCs were expressed as mean with SD. **(C)** Representative dot plots showing the gating strategies for intracellular cytokines in CD8^+^ T cells. **(D)** The percentages of IFN-γ, TNF-α, and IL-2 single-, double-, and triple-positive cells within CD8^+^ T cells from IM patients and HCs were expressed as mean with SD. Blue circle points represent HCs, and red circle points represent IM patients. *p < 0.05, **p < 0.01, ***p < 0.001.

The cytokine secretion of CD4^+^ and CD8^+^ T cells were assessed after stimulation with PMA/ionomycin. The percentages of IL-2 single-positive and triple-cytokine-positive (IFN-γ^+^ TNF-α^+^ IL-2^+^) CD4^+^ T cells were decreased significantly in IM patients (**Figures 3A, B**). However, the percentages of single cytokine positive and multifunctional CD8^+^ T cells (cytokine double positive and triple cytokine positive) were all decreased in IM group compared with that HCs (**Figures 3C, D**). These data suggested that although CD8^+^ T cells are expanded and activated rapidly during the early stage of EBV infection, they exhibited impaired cytokine secretion capability after re-stimulation *in vitro*.

### Monocyte and B Cell Subsets in IM Patients

**Figure 4:**
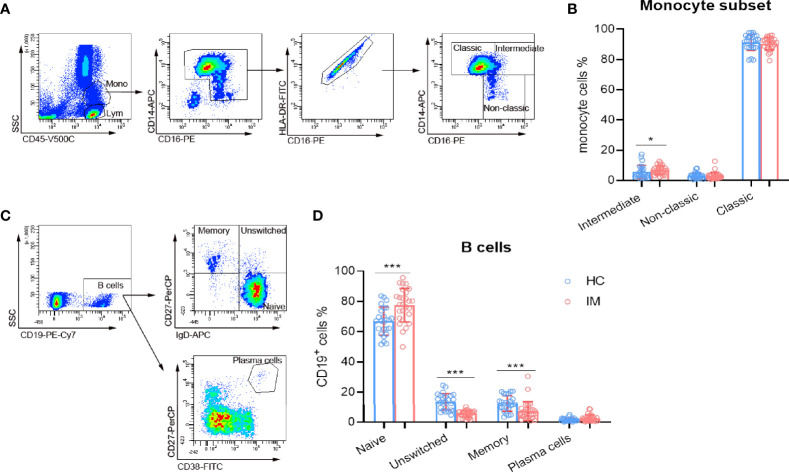
The subsets of monocytes and B cells. **(A)** Representative monocyte gating strategies in whole blood. CD45 *vs.* SSC plot: Broad selection of monocytes based on their SSC/CD45 properties. CD14 *vs.* CD16 plot: Gating to select monocytes based on their characteristic “┐” shape. CD16 *vs.* HLA-DR plot: Gating to select HLA-DR positive cells and remove NK cells. Selected monocytes redisplayed on CD16 *vs.* CD14 plot to gate the classical (CD14^++^ CD16^–^), non-classical monocyte (CD14^+^ CD16^+^), and intermediate (CD14^++^ CD16^+^) subsets. **(B)** The percentages of intermediate, non-classic, and classic monocytes from IM patients and HCs were expressed as mean and SD. **(C)** Representative flow dot plots showing the gating strategies of B cell subsets. CD19^+^ cells were gated and subclassified into Naïve (IgD^+^ CD27^−^), unswitched (IgD^+^ CD27^+^), and memory (IgD^−^ CD27^+^) cell populations. Plasma cells were gated as CD27^+^ CD38^high^ cell population. **(D)** The percentages of B cell Naïve, unswitched, memory, and plasma cells from IM patients and HCs were expressed as mean with SD. Blue circle points represent HCs, and red circle points represent IM patients. *p < 0.05, ***p < 0.001.

Monocytes exert innate immune response against viral infection, and the subsets of monocytes in IM were also assessed in the present study. Our results show that intermediate (CD14^++^ CD16^+^) subsets were found to be increased in IM patients, but not for classic (CD14^++^ CD16^−^) and non-classic (CD14^+^ CD16^+^) subsets. These results might be associated with the high inflammatory status of host immunity (**Figures 4A, B**). Meanwhile, EBV infects Naïve (IgD^+^ CD27^−^) B cells and promotes their differentiation into memory cells, or infects memory (IgD^−^ CD27^+^) B cells directly. The percentages of Naïve B cell subset were higher but with lower percentages of unswitched (IgD^+^ CD27^+^) and memory B cells (**Figures 4C, D**) in IM patients. These data suggested that the differentiation of B cells might be dampened during the course of EBV infection.

**Figure 5:**
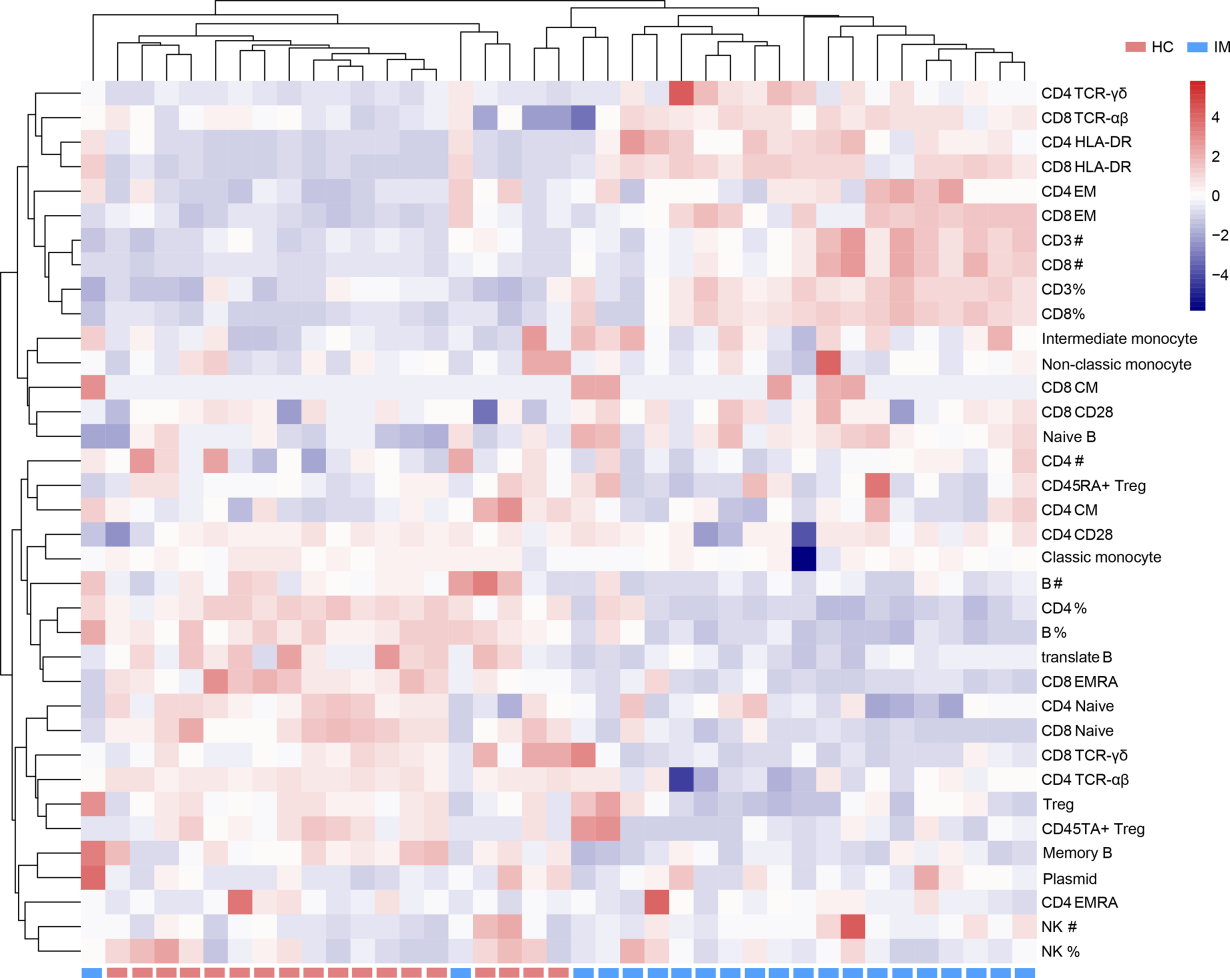
Immunophenotypes of mononuclear cells were shown in a heatmap with unsupervised clustering analyses.

Due to the heterogeneity of cell counts and immune marker expression among different individuals, a hierarchically clustered heatmap was developed for sample visualization (**Figure 5**). The overall analysis of immune signatures showed that the expression of HLA-DR, the proportion of EM subsets in CD4^+^ and CD8^+^ T cells, and the absolute counts of CD3^+^ and CD8^+^ T cells were increased, but the percentages of CD4^+^ T cells, B cells and Tregs, and naive subsets of CD4^+^ and CD8^+^ T cells were decreased in IM group compared with that HCs.

### Elevated Percentage of HLA-DR^+^ CD8^+^ T Cells Was Associated With the Clinical Severity of IM Patients

**Figure 6:**
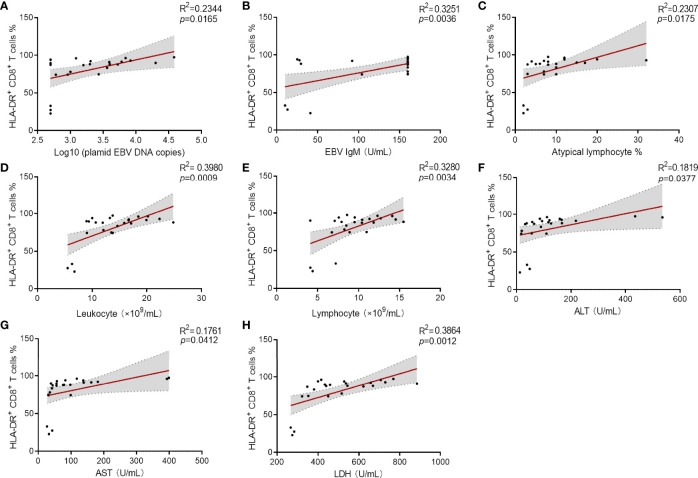
Correlation analysis between the percentage of HLA-DR^+^ CD8^+^ T cells and viral loaders and laboratory results. **(A–C)** Correlation between the percentage HLA-DR^+^ CD8^+^ T cells and plasmid EBV DNA copies (data were log10 transformed), EBV IgM levels and the percentage of atypical lymphocytes in peripheral blood smear. **(D, E)** Correlation between the percentages of HLA-DR^+^ CD8^+^ T cells and the counts of leukocytes and lymphocytes were shown. **(F–H)** Correlation between the percentages HLA-DR^+^ CD8^+^ T cells and the levels of ALT, AST, and LDH were shown. ALT, alanine aminotransferase; AST, aspartate aminotransferase; LDH, lactate dehydrogenase.

To investigate the association of immune signatures with clinical characteristics of IM, multivariate analysis was used, and the results revealed that the percentage of HLA-DR^+^ CD8^+^ T cells was the prominent factor for predicting the occurrence of IM (**Supplementary Table 1**). ROC curve analysis suggested that the percentage of HLA-DR^+^ CD8^+^ T cells was an effective marker in distinguishing IM patients from HCs, with an AUC of 0.997 (95% CI 0.905–1.000). The sensitivity and specificity of HLA-DR^+^ CD8^+^ T cell frequency at the best cutoff point (24%) were 95.24 and 100.0%, respectively. Then overall results of correlation analysis based on Spearman’s rank coefficient test were presented. Our data showed that percentage of HLA-DR^+^ CD8^+^ T cells was significantly associated with high plasmid viral load, EBV IgM level, percentage of atypical lymphocytes in peripheral blood smear, and abnormal blood routine and liver function results (**Figures 6A–H**). These data suggested that the elevated expression of HLA-DR in CD8^+^ T cells correlates well with the development and clinical severity of IM.

## Discussion

EBV infection is one of the most common infections in children and the main cause of IM (18). It is a major pathogen which is a serious threat to children’s health. EBV-IM is primarily transmitted through saliva contact, but it can also spread through blood, usually accompanied by an increase of atypical lymphocytes in peripheral blood. Although primary EBV infection is self-limited, it can be recurrent and associated with serious complications or poor prognosis in immune-deficiency patients (19). EBV infection is also one of the major causes of secondary HPS, which involves multiple organs and is life-threatening (20). Previous studies show that IM in childhood or adolescence had a 40% risk of subsequent diagnosis of depression, and excessive inflammatory is one key factor leading to depression (21, 22). Therefore, IM is an immunopathology condition, and its symptoms are caused by an excessive immune response to EBV infection (23, 24).

EBV infection initiates a polyclonal B cell proliferation and stimulates a vigorous immune response of CD8^+^ T cells to eradicate infected B cells. Previous studies showed that a huge predominance of mononuclear cells in circulating peripheral blood is EBV-specific CD8^+^ T cells in acute IM (25). In the present study, we observed noticeably increased counts of CD8^+^ T cells, but not for CD4^+^ T cells in response to EBV infection in a cohort of pediatric patients with IM compared with HCs, which was consistent with the previous studies (26). Further analysis revealed decreased percentages of Naïve subsets, but increased percentages of T_EM_ subsets for CD4^+^ and CD8^+^ T cells and T_CM_ for CD8^+^ T. These data demonstrated the rapid differentiation of Naïve T cells into effector and memory T cells with the stimulation of viral antigen. When effector T cells migrate to the site of inflammation to fight infection, memory T cells are distributed to survey and prevent the recurrence of disease. T_CM_ cells might lack effector properties, and T_EM_ cells could respond quickly to reinfection with lytic activity. Both CD4^+^ and CD8^+^ T_EM_ cells are characterized by rapid effector function in producing IFN-γ, IL-4, and IL-5 than T_CM_ cells upon antigenic stimulation (27). Therefore, much larger reservoir of memory CD8^+^ T cells and the balance between T_CM_ and T_EM_ are essential in immune control of persistent viral infections (26, 28, 29).

Immunophenotype analysis of T cells in children with IM showed significant activation of CD4^+^ and CD8^+^ T cells by detecting HLA-DR expression. Previous studies show that 34–54% of the HLA-DR^+^ CD8^+^ T cells in PBMCs were EBV-specific in febrile patients. The decline of HLA-DR^+^ CD8^+^ T cells was in parallel with reduction of EBV load (30). The upregulation of HLA-DR on CD8^+^ T cells was also reported in other virus infection, such as influenza, SARS, and Dengue (31–33). Therefore, effective expansion of reactive CD8^+^ T cells is needed for early protection against EBV infection. In addition, TCR is an important determinant of CD8^+^ T cell-mediated antiviral efficacy or immune-mediated pathogenesis (34, 35). TCR-αβ of CD8^+^ T cell responses to common viruses (influenza virus, cytomegalovirus) is favored for expansion due to the selection for optimal structural interaction (36). Our results indicated elevated TCR-αβ in CD8^+^ T cells, which correlated with virologic control and facilitated our understanding of how EBV-specific CD8^+^ T cells control EBV replication. Both CD8^high^ and CD8^dim^ T cells seemed to be highly activated, and the active phenotypes of CTL suggested the key role of these effector cells in eliminating EBV infection.

Our results showed an increased frequency of inhibitory molecules PD-1 and TIGIT on the surface of CD8^+^ T cells. Previous studies suggested that the upregulation of inhibitory receptors in CD8^+^ T cells correlates with dysfunction, and exhausted PD-1 expressing EBV-specific T cells lead to the loss of EBV control (37). In contrast, some studies showed that PD-1^+^ cells retain their proliferative, cytokine secretion, and cytotoxic capacities (38). Therefore, the functional heterogeneity of PD-1 might need further investigation. Moreover, we observed the decrease of multiple cytokines (IFN-γ, TNF-, IL-2) after stimulation of PMA/ionomycin, including single cytokine-positive and multifunctional CD4^+^ and CD8^+^ T cells. These data indicated that T cells, especially CD8^+^ T cells, become exhausted progressively in the course of EBV elimination in response to persistent antigen stimulation. Furthermore, multivariate analysis revealed that the percentage of HLA-DR^+^ CD8^+^ T cells was an independent prognostic marker for IM and significantly associated with the viral load and illness severity. The percentage of HLA-DR-expressing CD8^+^ T cell showed high sensitivity and specificity to distinguish IM patients from HCs. Therefore, HLA-DR^+^ CD8^+^ T cells could be used for the early screening, and help in the assessment of clinical severity of children with IM.

Several limitations of the present study should be mentioned. First, a larger number of children, especially those with immunocompromised status, are warranted to determine the alterations of mononuclear cell phenotypes in response to EBV infection. Second, antigen-specific T and B cell detection requires further investigation to claim the specific immune response in IM. Third, dynamic monitoring of immune signatures during EBV infection should be done for predicting the prognosis of IM.

## Conclusion

Collectively, this study assessed the numbers, phenotypes, and functions of lymphocytes and demonstrated the highly active phenotypes but impaired function of T cells during acute EBV infection. In addition, the change of CD8^+^ T cells was focused to be the prominent marker associated with the severity of children with IM. The evaluation of immunophenotypes not only extends further understanding of the pathogenesis of IM, but also may have valuable implications for guiding the diagnosis and therapy of the disease.

## Data Availability Statement

The original contributions presented in the study are included in the article/**Supplementary Material**. Further inquiries can be directed to the corresponding authors.

## Ethics Statement

The studies involving human participants were reviewed and approved by the ethical committee of Tongji Hospital, Tongji Medical College, Huazhong University of Science and Technology. Written informed consent to participate in this study was provided by the participants’ legal guardian/next of kin. Written informed consent was obtained from the individual(s), and minor(s)’ legal guardian/next of kin, for the publication of any potentially identifiable images or data included in this article.

## Author Contributions

YW and HH wrote the manuscript. YL, GT, RO, and MZ collected the materials of patients. YJ, TW, BY, and WW did the flow cytometry. XZ, JH, and MH analyzed the data. FW and SW helped revise the manuscript. All authors contributed to the article and approved the submitted version.

## Funding

This study was funded by the National Mega Project on Major Infectious Disease Prevention (2017ZX10103005-007).

## Conflict of Interest

The authors declare that the research was conducted in the absence of any commercial or financial relationships that could be construed as a potential conflict of interest.

## Publisher’s Note

All claims expressed in this article are solely those of the authors and do not necessarily represent those of their affiliated organizations, or those of the publisher, the editors and the reviewers. Any product that may be evaluated in this article, or claim that may be made by its manufacturer, is not guaranteed or endorsed by the publisher.
