# Supplementary figures and images for: HLA-DR Expression Level in CD8+ T Cells Correlates With the Severity of Children With Acute Infectious Mononucleosis
Source: Front Immunol. 2021 Nov 3;12:753290. doi: 10.3389/fimmu.2021.753290 (PMC8596082; doi:10.3389/fimmu.2021.753290)

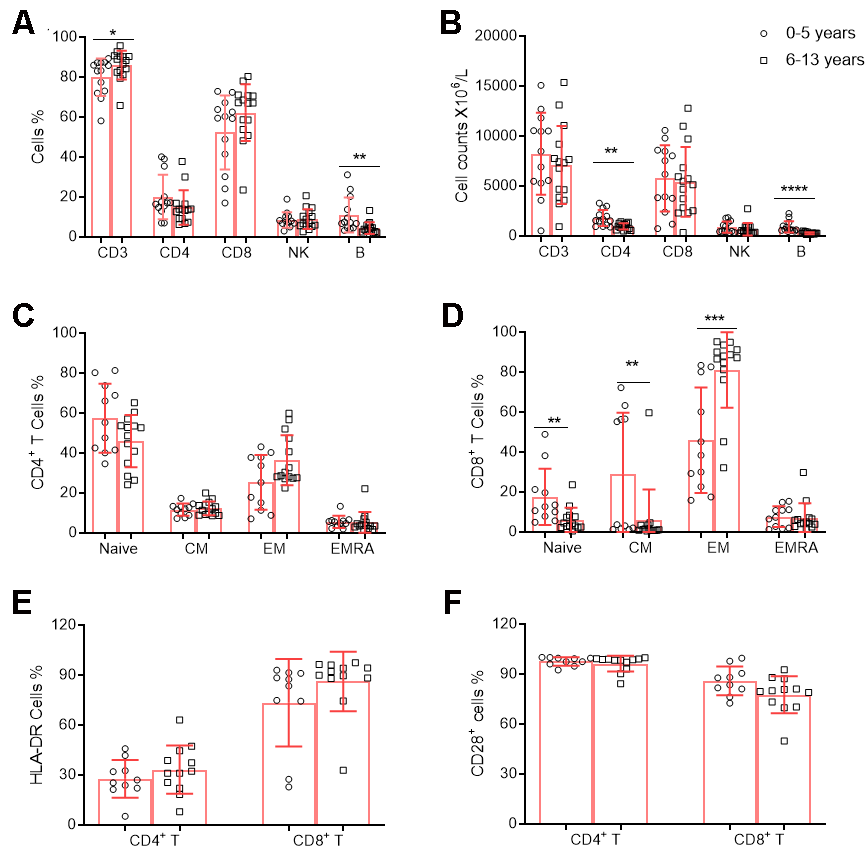

Supplement: Supplementary file 1 [file Image_1.tif]

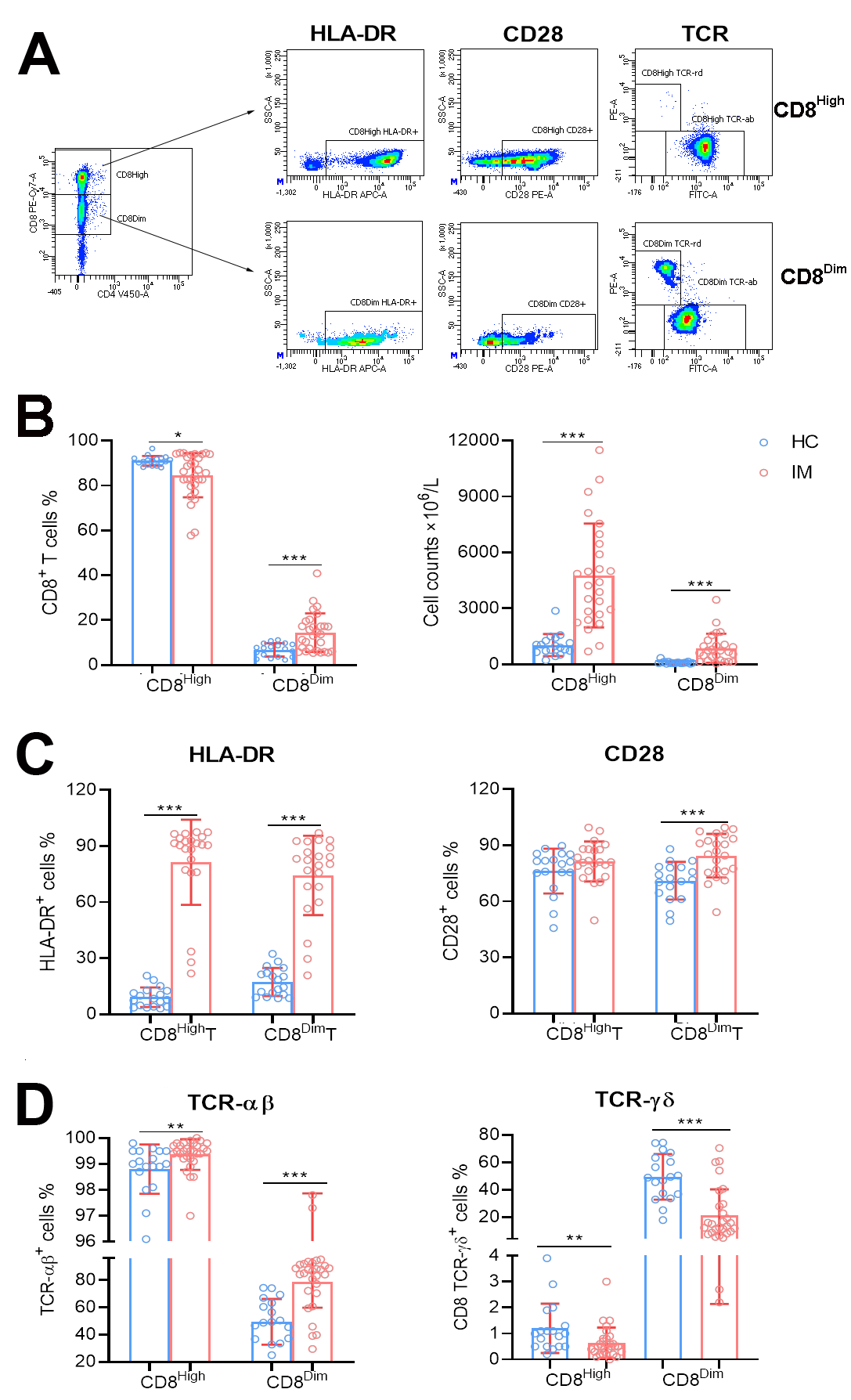

Supplement: Supplementary file 2 [file Image_2.tif]
