# Supplementary material for: HLA-DR Expression Level in CD8+ T Cells Correlates With the Severity of Children With Acute Infectious Mononucleosis
Source: Front Immunol. 2021 Nov 3;12:753290. doi: 10.3389/fimmu.2021.753290 (PMC8596082; doi:10.3389/fimmu.2021.753290)
Supplement: Supplementary file 3 [file Table_1.docx]

Supplementary table 1 Multivariable logistic regression analysis for prediction of infectious mononucleosis

| \| **Parameters** \| \| --- \| | HR | *p* value |
| --- | --- | --- | --- |
| CD3^+^ T % | 0.041 | 0.840 |
| CD3^+^ T counts | 0.018 | 0.892 |
| CD3^+^ CD4^+^ T % | 0.662 | 0.416 |
| CD3^+^ CD4^+^ T counts | 0.025 | 0.875 |
| CD3^+^ CD8^+^ T % | 0.129 | 0.719 |
| CD3^+^ CD8^+^ T counts | 0.035 | 0.852 |
| CD3^-^ CD16/56^+^ NK % | 0.102 | 0.749 |
| CD3^-^ CD16/56^+^ NK counts | 0.002 | 0.967 |
| CD19^+^ B % | 1.673 | 0.196 |
| CD19^+^ B counts | 0.000 | 0.999 |
| CD28^+^ CD4^+^ T % | 0.383 | 0.536 |
| CD28^+^ CD8^+^ T % | 0.009 | 0.925 |
| HLA-DR^+^ CD4^+^ T % | 0.004 | 0.949 |
| **HLA-DR^+^ CD8^+^ T %** | **11.64** | **0.000** |
| Treg % | 0.014 | 0.906 |
| CD45RA^+^ Treg % | 0.061 | 0.804 |
| CD45RA- Treg % | 0.541 | 0.462 |
| Intermediate monocyte % | 0.002 | 0.966 |
| Non-classic monocyte % | 0.675 | 0.411 |
| Classic monocyte % | 1.246 | 0.264 |
| Naïve B % | 0.012 | 0.914 |
| Unswitched B % | 1.274 | 0.259 |
| Memory B % | 0.012 | 0.913 |
| Plasma cells % | 0.034 | 0.853 |
